# Exploring the subjective experience of the “rubber hand” illusion

**DOI:** 10.3389/fnhum.2013.00659

**Published:** 2013-10-23

**Authors:** Camila Valenzuela Moguillansky, J. Kevin O'Regan, Claire Petitmengin

**Affiliations:** ^1^Laboratoire Psychologie de la Perception – CNRS UMR 8158, Université Paris DescartesParis, France; ^2^Instituto de Sistemas Complejos de ValparaísoValparaíso, Chile; ^3^Télécom École de Management, Institut Mines-TélécomParis, France; ^4^Archives Husserl, École Normale SupérieureParis, France

**Keywords:** rubber hand illusion, sense of body ownership, elicitation interview, micro-genesis, first-person methods, subjective experience

## Abstract

Despite the fact that the rubber hand illusion (RHI) is an experimental paradigm that has been widely used in the last 14 years to investigate different aspects of the sense of bodily self, very few studies have sought to investigate the subjective nature of the experience that the RHI evokes. The present study investigates the phenomenology of the RHI through a specific elicitation method. More particularly, this study aims at assessing whether the conditions usually used as control in the RHI have an impact in the sense of body ownership and at determining whether there are different stages in the emergence of the illusion. The results indicate that far from being “all or nothing,” the illusion induced by the RHI protocol involves nuances in the type of perceptual changes that it creates. These perceptual changes affect not only the participants' perception of the rubber hand but also the perception of their real hand. In addition, perceptual effects may vary greatly between participants and, importantly, they evolve over time.

## Introduction

The “rubber hand” illusion (RHI) is an experimental paradigm that has been widely used in the past 14 years to study a fundamental aspect of the sense of the bodily self: “the sense of body ownership” (e.g., Botvinick and Cohen, 1998; Ehrsson et al., 2004, 2005, 2007; Tsakiris and Haggard, 2005; Costantini and Haggard, 2007; Tsakiris et al., 2007a,b; Longo et al., 2008; Moseley et al., 2008a; Capelari et al., 2009; Kammers et al., 2009; Schütz-Bosbach et al., 2009; Lewis and Lloyd, 2010; Tsakiris, 2010).

In this experimental paradigm a person watches a rubber hand being stroked in the same location and at the same time as his or her own hidden hand. After a few minutes, most people get the curious impression that the rubber hand belongs to them. There are two requisites for this illusion to work: first, the rubber hand and the real hand have to be stroked at the same time and in the same place (synchronous stroking); and second, the rubber hand has to be placed in an anatomically plausible position. Generally, if the real hand and the rubber hand are not stroked synchronously (asynchronous condition), or if the rubber hand is not stroked (non-stroking condition) then the illusion is not induced. For this reason, these situations have been used as control conditions in several studies. The two most common measures used to evaluate the illusion are the displacement of the felt position of the hidden index finger in the direction of the rubber hand, termed “proprioceptive drift,” and the responses to a questionnaire.

Previous studies in the literature have assumed that only the synchronous condition causes a modification in the sense of body ownership, whereas both the non-stroking and asynchronous conditions have been assumed to be neutral. We realized that despite the vast number of studies that have used the RHI in the past 14 years, very few have sought to investigate the nature of the experience that the RHI supposedly evokes, more particularly whether this illusion involves “disownership” of the real hand in addition to ownership of the rubber hand. Fewer still have been the studies that have questioned whether the conditions used as controls are effectively neutral with respect to inducing some kind of illusion. To our knowledge, the only two studies that have assessed this issue directly are those of Longo et al. (2008) and Lewis and Lloyd (2010). Longo and collaborators used a psychometric approach to investigate the nature of the experience of embodiment using the RHI as a model. This approach involved the factor analysis of a 27-item questionnaire using Principal Components Analysis (PCA). This study identified certain aspects of the experience that characterize the synchronous and asynchronous stroking conditions in the RHI paradigm, and particularly the fact that the asynchronous condition might involve a sense of ownership of the rubber hand. Lewis and Lloyd (2010) interviewed participants who underwent a RHI-type procedure, and used interpretative phenomenological analysis to explore their experience. In their protocol participants underwent a sequence of three consecutive periods of stroking as follows: one synchronous, one asynchronous, and then a second synchronous. The authors used this approach to characterize the experience of the RHI, considering these three periods as a continuum. Among other results, they found that the asynchronous condition did induce some form of the illusion. However, they did not compare the illusory experiences in the synchronous and asynchronous conditions. Moreover, none of these studies assessed the experience of the non-stroking condition.

For these reasons we considered it necessary to carry out a fuller examination of the phenomenology of the RHI, including all of the generally used stroking conditions. An additional question was derived from a pilot experiment that we carried out.

In this pilot experiment, conducted with individuals who were trained in a first-person technique called “elicitation interview” which will be presented in the Methods section, one of the participants described a dissociation between the hand (her own or the rubber hand) where she felt the contact of the paintbrush and the hand (her own or the rubber hand) where she felt the contact of the table underneath. As the illusion unfolded the participant experienced a unification between the hand that was being touched by the paintbrush and the hand that was touching the table, suggesting also the presence of different stages in the emergence of the illusion, as illustrated by the following extract of her description:

“I really *have the tactile sensation of my hand lying on the table*, in contact with the table and then, *I feel the contact with the brush which I can't see*.”^1^.

*“Progressively the tactile sensation transfers itself into the rubber hand* … you see it's bizarre, because I'm between the two, I have the tactile sensation of the paintbrush here (indicating the rubber hand), but I still feel my real hand, the heat of it, *the contact with the table is on the right* [with the real hand]*… it's bizarre, I have this partial illusion.”*

“Something changed, something changed in the sensation, and then after a while, well, I felt the… I felt *not only the brush on my hand here, there*, [on the rubber hand], *but I also felt the contact of my fingers with the piece of cardboard* [under the real hand].”

“The illusion *will be complete when I have everything* [the contact of the fingers with the table and the contact of the brush], *in the rubber hand”*

We related these two aspects of touch to the touching/touched [“touchant”/”touchée”] distinction used by Merleau-Ponty while discussing the particularities of the experience of “being a body” (2009 [1945], pp. 121–122). When my left hand is *touching* my right hand, my right hand is for my left hand an “object” made of skin, muscles and bones. At the same time, I feel my right hand *touched* by the fingers of my left hand. This double sensation of touching and being touched is experienced when the hands touch each other, but also in any tactile sensation: whatever the object, I may have the feeling of touching it or being touched by it. This alternation is characteristic of the feeling of recognizing our body as our own—of the feeling of body ownership. The feeling of *being touched* may be considered as the passive component of touch, the feeling of *touching* as its active component.

In the context of the RHI, we consider the feeling of being touched by the paintbrush as the passive aspect of touch, and the feeling of touching the table as its active aspect. Although the paintbrush is moving, its contact is indeed received passively by the hand. And although the hand is static on the table, it is potentially able to explore the table, if not by real movements, at least through slight changes in pressure or even perhaps imagined movements.

So far, studies on the RHI have assessed whether the passive aspect of touching, the *being touched*, is affected by the illusion (as illustrated by the statement classically used in the RHI questionnaire: “It seemed as if I was feeling the touch of the paintbrush in the location where I saw the rubber hand”) but they have never assessed the active or *touching* aspect of the situation (the location of the sensation of touching the table). Therefore, we considered that it would be interesting to investigate whether the passive and the active components of touch are both transferred in the RHI, or whether a dissociation may occur. In addition, we considered it relevant to determine whether there are different stages in the perceptual changes linked to the illusion.

Thus, the main objectives of this study are (a) to assess whether the different stroking conditions in the RHI protocol induce ownership of the rubber hand and/or disownership of the real hand, (b) to investigate whether the RHI involves a transfer of both the passive and the active components of touch or if the two are dissociated; and (c) to determine whether there are different stages in the emergence and evolution of the illusion.

In order to do so, we used the “elicitation interview”^2^ technique (Vermersch, 1994/2011), a technique stemming from phenomenology. Using this methodology we gathered detailed descriptions of participants' experiences during the classical protocol of the RHI. The different conditions of stroking were: “synchronous stroking,” which corresponded to synchronous stroking of the rubber and the real hand; “asynchronous stroking,” in which the rubber and the real hand were stroked at a different place in a given time; and the “non-stroking,” in which the rubber hand was not stroked. On the basis of on an analysis of these descriptions, we identified the features that characterized each participant's experience in each stroking condition. We also identified different phases in the participants' experiences of the RHI. Regularities in the participants' experiences allowed us to highlight a generic structure of the emergence of the illusion and the main features of the experience in each stroking condition.

The aim of this study is above all descriptive; the results do not have a quantitative meaning, and therefore no statistics are involved.

In what follows, the methodology of the study, the results of the analysis of the interviews, and a discussion are presented successively.

## Methods

### Participants

Following approval by a local Ethics Committee, the experiment included 10 participants (9 right-handed; five females; mean age 31.7 ± 2.1 years) who were informed of the experimental procedure but not of the hypothesis of the study.

### Design of the experiment

The experiment had one independent variable, which was the type of stroking condition. The stroking conditions were the *synchronous* condition, in which the rubber and real hand were stroked synchronously; the *asynchronous* condition, in which the rubber and the real hand were stroked at a different place at each given moment; and the *non-stroking* condition, where the participant looked at the rubber hand but tactile stimulation was given only to the real hand, and not the rubber hand. The order of presentation of the conditions was counterbalanced between the participants.

Interviews were conducted during and after the different periods of stroking, as prescribed by the elicitation interview technique.

### Apparatus

**Figure 1:**
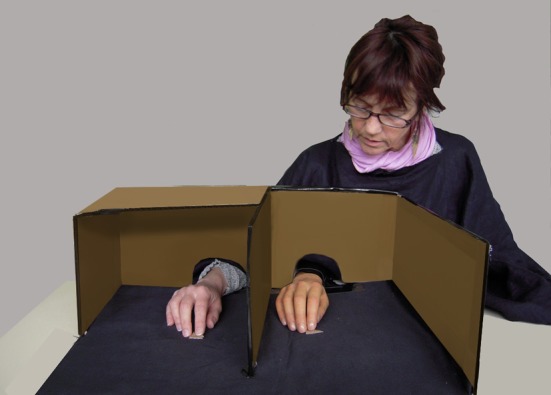
**Experimental set up, seen from experimenter's viewpoint.** Participants placed their right hand inside the cardboard box; they placed their index finger on a mark. They could see the rubber hand (here on the right part of the Figure) but not their real hand.

In order to induce the RHI, we used a specially constructed cardboard box and a prosthetic hand (Figure 1). The prosthetic hand was located 20 cm to the right of the right hand. A cardboard cover was placed on top of the box. When the cover was removed, the participant could see the rubber hand, and when the cover was placed over the box, the participant could not.

### Procedure

Participants sat in front of a table with their right, stimulated arm placed through a hole cut in the front of the cardboard box. The cover was removed and the rubber hand became visible to the participant. The stroking stimulation period then began (one of the three stroking conditions described above). During the stroking period, the participants were asked to describe their experience using the interview technique explained in the following section. The length of the period of stroking varied depending on the evolution of the interview. At the end of each stroking stimulation period, the cover was again placed over the box. Each participant underwent a total of six experimental blocks, two for each stroking condition. The order of the conditions was counterbalanced between participants. The experiment lasted ~70 min.

### Interview technique

The “elicitation interview,” first introduced by Pierre Vermersch (1994/2011, 1999) and developed in the field of cognitive science by Claire Petitmengin (1999, 2006), is a technique that attempts to guide a person to recall a given experience, examine it, and describe it with great precision. Originally designed to study the cognitive processes involved in learning, the technique was then incorporated into the neurophenomenological program proposed by Francisco Varela (1996) and since then has been used and tested by a growing number of researchers in the cognitive (e.g., Lutz et al., 2002; Petitmengin et al., 2013), clinical (Petitmengin et al., 2007), therapeutic (Katz, 2011) and managerial (Remillieux, 2009) fields.

#### Gathering of the data

In the present study, the gathered descriptions were collected “in real time,” during induction of the illusion, the interviewees describing their experience as it unfolded. Other descriptions were given after the end of the stroking period.

Once the stroking period started, participants were asked to describe what they perceived or felt whenever they wanted to. With their agreement, participants' descriptions were recorded. In cases where participants spontaneously expressed a particular feeling, either through words, exclamations or gestures, they were questioned about what they had just said or expressed. In cases where they did not spontaneously express any particular feeling, questions such as “And now, what do you feel?” or “And now what's happening?” or “Do you feel anything toward the rubber hand?” were used to trigger participants' descriptions. In the cases where participants preferred not to verbalize during the stroking period, they were led to evoke the stroking moment immediately after it was finished, and questions were asked in order to explore their sensations.

In order to identify whether there was a dissociation between the passive and active components of touch, in the case that participants did not refer spontaneously to this issue, we asked them systematically pre-established questions. The questions concerned (a) the location of the tactile sensation produced by the paintbrush (whether on the rubber hand or on the real hand); (b) the location of the tactile sensation produced by the contact with the table (whether on the rubber hand or on the real hand).

The following paragraph shows an example of an interview during the synchronous stroking condition. For a detailed description of the methodology of interview and analysis, see Petitmengin (1999, 2006) and Valenzuela-Moguillansky (2012).

– Now I have the impression that the hand I see is my own hand.
– How do you know this?
– Because I just moved my fingers, and I said to myself: “Why does it not move, this hand?”
– Can you come back to this moment? What happens when you move your fingers?
– (Silence…) I can feel the cardboard under my finger, but I see that hand does not move, I have the feeling that she does not answer.
– When you have the feeling that she does not answer, what do you feel?
– I feel my arm like a dead weight, something very heavy. This is actually quite unpleasant.
– How is this feeling of dead weight, what is it like?
– Uh… I have the impression that my arm is very heavy. Almost a kind of paralysis, which goes up slowly.
– Which goes up slowly. Until where does it go up slowly?
– It goes up a little in the shoulder… my shoulder is like numb. And I say to myself: “Shit, now my hand is paralyzed, what am I going to do, how will I get back my hand there?”

#### Analysis of the data

Once the interview had been transcribed, the first step of the analysis consisted in setting aside the anecdotal descriptions and comments and in selecting the relevant information related to the experience itself. The second step consisted in identifying both the *diachronic* and *synchronic* structure of the participant's experience. The diachronic structure corresponds to the successive phases of the experience, while the synchronic structure corresponds to the characteristics of the experience at a given phase. In this study, the diachronic analysis consisted in identifying the participant's perceptual changes that marked different phases of the experience in a given stroking condition. This process allowed the identification of the diachronic structure of the participant's experience in a given stroking condition. We then looked at the experiential categories that characterized each of the phases previously determined, identifying the synchronic structure of each phase for a given participant in this stroking condition. In this way we created an individual representation of the structure of the experience of each stroking condition for each participant. We repeated this procedure for each participant and then compared the individual structures, looking for invariants across different participants. Based on the invariants, we constructed the generic structure of the experience for each stroking condition. A more detailed description of the analysis of the data is provided in the Appendix and in Valenzuela-Moguillansky (2012).

## Results

**Figure 2:**
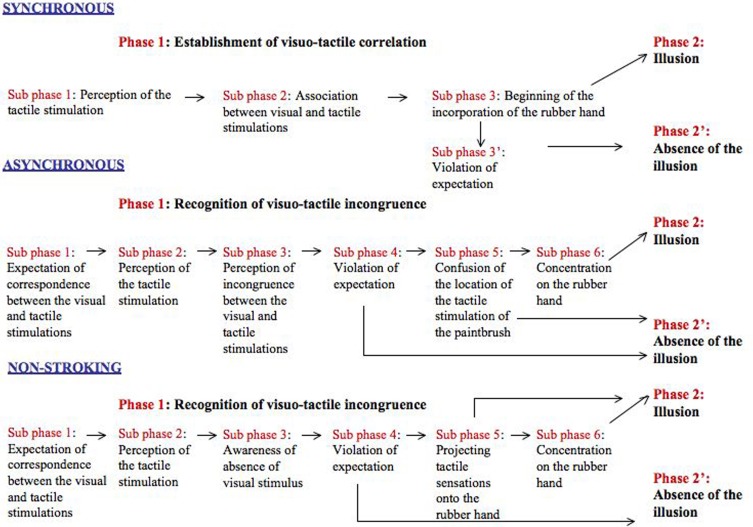
**Generic diachronic structure of each visuo-tactile condition.** The name of each visuo-tactile condition appears in blue. Black arrows indicate sequential order of the phases and sub phases.

The identified generic structure of the RHI experience for each stroking condition is made up of a generic diachronic structure (Figure 2) and a generic synchronic structure. The generic diachronic structure is made up of two phases. The first phase of each stroking condition is composed in turn by a series of operations or “sub phases” that are organized in a sequential order. Some participants described certain phases in more detail than others, and some concentrated their description on only one phase. The generic synchronic structure is made up of several experiential categories that characterize Phase 2 and 2′. These experiential categories are not organized in a sequential order (Table 1).

**Table 1:** **Summary of the experiential categories corresponding to Phases 2 and 2' in the three stroking conditions**.

**Synchronous**	**Asynchronous**	**Non-stroking**
**8/10**	**7/10**	**3/10**
**PHASE 2: ILLUSION**		
Feeling of ownership of the rubber hand	Feeling of ownership of the rubber hand	Feeling of ownership of the rubber hand
Disregarding the real hand	Feeling of ownership of the real hand	Delocalization of the real hand
Delocalization of the real hand	Attribution of real hand features to the rubber hand	Attribution of real hand features to the rubber hand
Disruption of proprioception	Feeling a “numbed” hand	Acquisition of rubber hand features
Attribution of real hand features to the rubber hand	Feeling a phantom sensation	Feeling of having a prosthesis
Acquisition of rubber hand features		
**PHASE 2′: ABSENCE OF THE ILLUSION**		
Absence of feeling of owning the rubber hand	Absence of feeling of owning the rubber hand	Absence of feeling of owning the rubber hand
Feeling of ownership of the real hand	Feeling of ownership of the real hand	Feeling of ownership of the real hand

The second row indicates the number of participants out of the total that experienced the illusion in each stroking condition. It is important to note that not all the participants that experienced the illusion in a given stroking condition described all the experiential categories.

In this section we present the generic structure of each stroking condition. The sub phases and experiential categories are accompanied by one or more descriptive statements that illustrate them. At the end of each descriptive statement, the initials of the participant are indicated in parentheses. Questions from the interviewer appear in bold. Key parts of the descriptive statement are in italics.

### Synchronous stroking

#### Phase 1: establishment of the visuo-tactile correlation

In this phase participants felt the tactile sensation from the paintbrush and established an association between the tactile sensation and what they saw. We identified four “sub phases” that characterize the diachronic structure of Phase 1.

***Sub phase 1: perception of the tactile stimulation.*** This sub phases corresponds to participants' description of feeling the movement of the paintbrush on their hand.

“… [I feel] *the tickles of the paintbrush* in my right hand” (C.G.)

***Sub phase 2: association between visual and tactile stimulations.*** This sub phases includes participants' description of perceiving the correspondence between what they saw on the rubber hand and what they felt in their own hand.

“*My sensation is exactly the same as what I see*, (…) Even though I know that it's not my hand” (V.C.)

One participant described having the impression of being able to predict the tactile sensation from the visual input. We believe that the “impression of prediction” illustrates a process of association between visual and tactile stimulation.

“I have a sensation, as if I am watching the paintbrush coming close to each part of the hand and in certain way *I can predict what I will feel*.” (C.V.)

***Sub phase 3: beginning of the incorporation of the rubber hand.*** This sub phase groups together the passages in which participants referred to the experience of owning the rubber hand but in a way that suggested that they were “in the process of” integrating the rubber hand—that the feeling of ownership was not yet complete. This can be observed mainly through two features of the participants' speech: the use of the conditional tense, and the suggestion that the experience is beginning or is not yet there but that it could come in the near future.

“I have the impression that if you keep going for another ten minutes, *I could believe* that the other hand is my hand, actually…” (G.R.)

“Actually the longer it lasts the more I have exactly this impression, that it [the illusion] *is starting*, well the fake hand, I have the impression that it's mine.” (H.M.)

Regarding the location of the tactile sensation produced by the paintbrush and the location of the tactile sensation produced by the contact with the table, in this phase some participants described feeling the tactile sensation produced by the paintbrush on the rubber hand and all of them described feeling the tactile sensation produced by the contact with the table under their real hand.

***Sub phase 3′: violation of expectations.*** This sub phase includes participants' descriptions of perceiving incongruence between what they were seeing and what they were feeling. This feeling was accompanied by a feeling of strangeness or surprise. Although the stroking of the rubber and real hands was meant to be synchronous and equal, because it was performed manually, presumably it was not strictly identical, and small asynchronicities were present for all participants. Only two participants described this as a salient feature of their experience—these were the participants that did not experience the illusion in the synchronous stroking condition.

“*It's crazy!* It's like if it gets a bit detached now, or *I don't know if you are doing it a little bit shifted or exactly at the same time*, but it's almost like the sensation (tactile) is a bit before what I am seeing … ” (C.V., 173)

“And there's something funny … sometimes I feel the coldness of the metal, but I don't see the metal touching here (…) and it makes me think, *it makes me aware that this [the rubber hand] is not my hand*.” (M.W., 45)

In one participant, the awareness of incongruence between the stroking of the rubber and real hands triggered a chain of realizations which ended with the discovery that he had implicitly incorporated the rubber hand.

“it [the incongruence between what he sees and what he feels] makes me think that, it makes me become aware that this [the rubber hand] is not my hand but this means that (laughs), *that if this makes me aware that this one is not my hand, before they were confused, that's what I really feel”* (M.W., 49)

#### Phase 2: illusion

This phase corresponds to the illusion itself. We identified six experiential categories that characterize this phase. From participants' descriptions we could not identify a generic sequential order between them. Thus, they characterize the synchronic structure of Phase 2 and not its diachronic structure.

***Feeling of ownership of the rubber hand.*** The presence of the illusion is generally determined by the participants' spontaneous report of the feeling that the rubber hand belongs to them. This category groups together participants' descriptions of such a feeling of ownership.

“*I really have the impression that the rubber hand is my hand*.” (D.L.)

***Disregarding the real hand.***

“It's pretty bizarre because *I forget that I have this right hand*.” (V.C.)

***Delocalization of the real hand.*** This subcategory includes descriptions of the delocalization of the real hand. The descriptions regarding the delocalization of the tactile sensation produced by the paintbrush were in most cases spontaneous. The descriptions regarding the delocalization of the tactile sensation produced by the contact with the table was spontaneous only for one participant. In the case of the other participants the location of the tactile sensation produced by the contact with the table was assessed through the pre-established questions.

Thus, we identified that the delocalization of the participants' real hand could be evaluated in terms of the following points of reference:

*Location of the tactile stimulation with respect to the hand*.

“That is … the position, well, … *the place where I feel the brush is the place where I see the rubber hand*.” (D.L.)

*Location of the hand with respect to the table*.

“*I have the impression that my hand is on the table here* (indicating the rubber hand)…” (H.M.)

*Location of the hand with respect to the rest of the body*.

*“I feel as if it (the rubber hand) was placed as my hand, in the prolongation of my arm*.” (D.L.)

***Disruption of proprioception.*** This subcategory corresponds to one participant's description of not knowing the position of her real hand. She assimilated the position of her hand to that of the rubber hand.

“I am very curious to see *whether my hand is in the same position as this one (the rubber hand) because to me it seems that it's at exactly the same position* but no ….” (I.P.)

***Attribution of real hand features to the rubber hand.*** This category includes participants' description of attributing features of the real hand to the rubber hand. There were different kinds of features that were attributed to the rubber hand. We have grouped them into the following categories: agency, life, sensitivity and texture and warmth.

***Acquisition of rubber hand features.*** In this category are participants' descriptions of experiencing their hands as having acquired the features of the rubber hand. The features are further classified into different subcategories: texture, marks and color.

#### Phase 2': absence of the illusion

This is the final phase of the experience of two participants who stated that they did not experience a feeling of ownership toward the rubber hand.

***Absence of feeling of owning the rubber hand.*** This category includes statements of not feeling ownership of the rubber hand.

“*I still don't feel that the rubber hand is my hand*, I don't yet have this feeling of ownership.” (C.V., 181)

***Feeling of ownership of the real hand.*** This category includes participants' descriptions of feeling their real hand as present. The presence of participants' real hand was expressed by referring to the localization of their hand. This was done in terms of three points reference: localization of the tactile stimuli with respect to the hand; localization of the hand with respect to the table; and localization of the hand with respect to the rest of the body. In this case neither the active aspect of touch nor the passive one are transferred to the rubber hand.

***Location of the tactile stimulation with respect to the hand.***

**“Where do you feel the paintbrush? On the rubber hand or on your real hand?**

I don't know (laughs) no, *still on my hand I think.”* (M.W., 54)

*Location of the hand with respect to the table*.

**“Where do you localize the contact with the table? Under the rubber hand or under your hand?**

*Under my hand.*” (M.W., 61)

*Location of the hand with respect to the rest of the body*.

“B*ut I still feel the connection of my hand with the rest of my body, ultimately and this sensation has not been transferred to the rubber hand*.” (C.V., 200)

### Asynchronous stroking

Seven out of ten participants had some kind of illusory experience in the asynchronous condition.

#### Phase 1: recognition of visuo-tactile incongruence

As in the synchronous condition, this phase was the one wherein participants experienced the tactile sensation of the paintbrush. Contrary to the synchronous condition, in the asynchronous condition, tactile sensations did not correspond with what participants were seeing. This is why, instead of establishing an association between the visual and tactile stimulation, at this stage most participants recognized the incongruence between visual and tactile stimulation. We identified four “sub phases” that characterize the diachronic structure of Phase 1.

***Sub phase 1: expectation of correspondence between the visual and tactile stimulations.*** This sub phase includes participants' expectation of correspondence between visual and tactile stimulations.

“*I see the brush and so I expect to feel the physical response … ”* (G.R.)

***Sub phase 2: perception of the tactile stimulation.*** This sub phase includes participants' descriptions of the tactile sensation of the paintbrush on their real hand, without doing any mention of feelings of identification with the rubber hand or any strange sensation.

“I feel touch from a brush.” (H.M.)

***Sub phase 3: perception of incongruence between visual and tactile stimulations.*** This sub phase includes participants' descriptions of incongruence between what they were seeing and what they were feeling. This distinction was sometimes accompanied by a feeling of strangeness and sometimes not.

“*The stimulation is different well, different geographical zones that are stimulated…*.” (V.C.)

***Sub phase 4: violation of expectations.*** This sub phase includes the realization that the tactile stimulation does not correspond with the visual one. Generally this realization was accompanied by a feeling of strangeness and confusion.

“*Visually I expected to see on the rubber hand what I was feeling in my hand”* (D.L.)

“*I see the brush and so I expect to feel the physical response* and it's really weird not to feel it … ” (G.R.)

***Sub phase 5: confusion of the location of the real hand.*** This sub phase corresponds to participants' descriptions regarding the confusion of the location of location of the location of the tactile sensation elicited by the paintbrush and the location of their arm with respect to the rest of the body. Contrary to what happened in the synchronous condition, in the asynchronous condition, participants' descriptions suggested that the confusion in the location of their hand was a stage that leads to the emergence of the illusion and not characteristic of the illusion itself.

“Now there's a sort of confusion, because I, well, my brain sees that I can't have two hands, *and that I feel in two different places, I feel in my real hand and I have a little bit of an impression, only when the brush is touching, not when it doesn't touch the hand, that the rubber hand, not necessarily that it belongs to me, but that I feel something on it*.” (D.L.)

“I feel as *if my forearm was in the middle (between the real hand and the rubber hand), maybe not, maybe more on the side of my real hand in fact”* (D.L.)

***Sub phase 6: concentration on the rubber hand.*** This sub phase corresponds to participants' descriptions that indicate that concentrating on what is happening on the rubber hand leads to the illusion.

“*If I look at the visual contact between the paintbrush and the rubber hand, then it has simply become my hand”* (G.R.)

#### Phase 2: illusion

This is the phase where participants described having illusory sensations and feeling ownership of the rubber hand. The illusion was present to different degrees of intensity and with varying characteristics. A salient feature of this illusion in comparison to the one in the synchronous condition is the fact that some participants experienced a “phantom tactile sensation,” meaning that they felt the touch where they saw it (on the rubber hand) even though they were not being stimulated at that moment at the corresponding location.

***Feeling of ownership of the rubber hand.*** As in the synchronous condition, this category includes descriptions of ownership of the rubber hand. In the case of the asynchronous condition, the intensity of this feeling was more variable than in the synchronous condition.

“It is as if it was *a little bit my hand* [the rubber hand], as if it was a bit numbed” (J.A.)

*“It increased, it increased (the feeling of owning the rubber hand)*, because … what I feel now is where *you brush with the paintbrush, that's where I feel, woo! Yes! … the sound made by paintbrush is like* …

**Like what?**

Like as if I *were feeling it in my skin*.” (I.Q.)

***Feeling of ownership of the real hand.*** This category includes participants' descriptions of feeling the presence of their real hand and ownership of the rubber hand at the same time.

“It's very strange … *yes*, the rubber hand is still part of my body, but in a less obvious way and *my other hand becomes more present, but not yet in a definitive way*.” (I.P.)

**“And the table under your hand, do you feel it under your hand or under the rubber hand?**

*So under my real hand*, my physical hand at the level of the table.” (D.L.)

We can observe that, contrary to what happened in the synchronous condition, in the asynchronous condition there is no transfer of the active or passive aspects of touch during the illusion. As presented in the sub phase 5, participants' descriptions suggest a confusion regarding the localization of the tactile sensation produced by the paintbrush and in the location of the arm with respect to their body. However, participants' ability to localize their real hand with respect to the table does not seem to be disrupted.

***Attribution of real hand features to the rubber hand.*** As in the synchronous condition, this category includes participants' attribution of real hand features to the rubber hand.

*Movements*.

“I just felt that my hand made these involuntary movements, *and it's as if at any time the rubber hand could do the same thing* … ” (C.V.)

*Life*.

“It [the rubber hand] *began to acquire more life* (…) but it's not that it necessarily belonged to me, as an extension of my body” (C.V.)

***Feeling a “numbed” hand.*** This category is specific to the asynchronous condition and includes the reports of two participants who described having the feeling that the rubber hand belonged to them but as if it were numb.

“I feel like when *your hand is numbed, when you fall asleep* on your hand and you have to take it and move it around” (M.W.)

***Feeling a phantom sensation.*** This category is specific to the asynchronous condition and includes participants' descriptions of feeling a phantom tactile sensation on their real hand at the location where they see the paintbrush touching the rubber hand, even though they know that the paintbrush is touching a different location on their real hand. Here it is important to recall that the asynchronous condition involves the fact that the rubber hand that the person sees and the real hand that the person does not see are stroked at different places at given moment. This category was present only in participants who underwent the synchronous condition before the asynchronous condition, suggesting that the order of the conditions has an impact on what is experienced in each condition.

“So now really right away I have, *as if I felt the ghost [fantasme] of the brush*…That's it and so *I feel it, I feel it… not with the same force*… but the illusion of, well the instinct of feeling is so strong that it creates a sensation. *So in the end it's as if I felt the ghost [fantasme] of your brush*, you see what I mean?” (G.R.)

#### Phase 2': absence of the illusion

This phase corresponds to the final stage of the experience for participants who did not experience the illusion in the asynchronous condition.

***Absence of feeling of owning the rubber hand.***

“… now *I feel my hand perfectly and the illusion doesn't work*.” (V.C.)

***Feeling of ownership of the real hand.*** As in the synchronous condition, participants who did not experience the illusion felt the presence of their hand mainly through its localization according to three points of reference: the localization of the tactile stimuli with respect to the hand; the localization of the hand with respect to the table; and the localization of the hand with respect to the rest of the body.

*Location of the tactile stimuli with respect to the hand*.

“*I feel it clearly, that is, I mean, I feel exactly that there's a little tiny bit of pressure from the brush on the hand*.” (V.C.)

*Location of the hand with respect to the table*.

“Indeed *the hand will enter a little bit, into a little bit closer contact with the table*, so now I feel also from underneath that *it actually is my fingers that are in contact with that table*.” (V.C.)

*Location of the hand with respect to the rest of the body*.

“*I could say that it climbs up the arm*, because it doesn't really come from the arm, but I feel perfectly that it's mine.” (V.C.)

### Non-stroking condition

Three out of the ten participants had some kind of illusory experience in the non-stroking condition. For two of the three participants who experienced the illusion, the non-stroking condition was presented after the synchronous condition. For one of the three participants who experienced the illusion, the non-stroking condition was the first of the experiment.

Some of the experiential categories seen here are shared with the previous conditions. In those cases their definitions are not repeated. Differences are specified where necessary.

#### Phase 1: recognition of visuo-tactile incongruence

As in the asynchronous condition, this is the phase in which participants became aware of the incongruence between what they were seeing and what they were feeling. In the case of the non-stroking condition the incongruence is even more evident, because the rubber hand is not being stroked at all.

***Sub phase 1: expectation of correspondence between the visual and tactile stimulations.*** As in the asynchronous condition, this sub phase includes participants' expectation of correspondence between visual and tactile stimulations. In the case of the non-stroking condition this phase is implicit, meaning that participants did not directly express the expectation, but the fact that participants expressed a violation of what they had been expecting implies that this expectation had been present.

***Sub phase 2: perception of tactile stimulation.*** As in the previous conditions, this sub phase includes participants' descriptions of the contact of the paintbrush with their hand without particular reference to ownership or any other strange sensation.

“Okay… *for now I'm able to localize it, now it's on the thumb, now on the index finger* (…) it's pleasant,…” (D.L.)

***Sub phase 3: awareness of absence of visual stimulus.*** This sub phase simply includes descriptions of the absence of the paintbrush on the rubber hand.

“Well you see the fake hand, *but you simply don't see the brush”* (V.C.)

“*The fact that there's no visual stimulation, just touch.* So actually I concentrate more on the touch on my hand and less on the visual side. So actually now, the rubber hand doesn't at all seem to be me. So I definitely make the distinction on that problem.” (H.M.)

***Sub phase 4: violation of expectation.*** This sub phase includes description of the violation of an expectation of correspondence between the tactile and visual stimuli. Sub phase 1 (Expectation of correspondence between the visual and tactile stimulations) is inferred from the presence of this category.

“It's as if *I should feel the brush on the fake hand*, but in fact I feel it somewhere else” (G.R.)

***Sub phase 5: projecting tactile sensations onto the rubber hand.*** This sub phase includes participants' descriptions of projecting the tactile sensation that they were feeling on their real hand onto the rubber hand.

“I mean, for instance *I was trying to follow with my eyes, to look at the same places that you were touching with the paintbrush on my hand, but on the rubber hand*” (C.V.)

***Sub phase 6: concentration on the rubber hand.*** This sub phase corresponds to two participants' descriptions of concentrating on the sensation elicited by projecting the tactile sensation of the paintbrush onto the rubber hand.

“Well *I was concentrating* on that strange sensation.” (G.R.)

#### Phase 2: illusion

This is the phase in which participants experienced ownership of the rubber hand and described “unusual feelings.” Only three participants experienced this phase. In the case of two participants, the description of the experience included experiential categories similar to those associated with the illusion in the synchronous and asynchronous conditions. In these cases we called this phase “Standard Illusion.” In the case of one participant, the illusion was expressed in a particular way that we will describe as a subtype of illusion called “Feeling a Prosthesis.”

***Feeling of ownership of the rubber hand.***

“*I start to feel that it was mine* [the rubber hand], I mean, *I could even talk about a third limb*, no? Because I'm feeling *that something is happening there* [on the rubber hand]” (C.G.)

**“So, the feeling that the rubber hand is your hand remains?**

*Yes, no doubt, yes*.

**And is it more or less intense than before [synchronous condition]?**

*More intense”* (I.Q.)

***Delocalization of the real hand.***

**“And the table under your hand, where do you feel it? Under the rubber hand or under your hand?**

Now I feel it *more under my rubber hand* … of MY rubber hand! (Laughs)” (J.A.)

***Attribution of real hand features to the rubber hand.***

*Movement*.

“It's crazy, I feel as if the thumb of this hand [rubber hand] *had moved*” (I.Q.)

*Life*.

“What *I feel now is as if this [the rubber hand] was my hand*, but as if it was a little *bit swollen, I mean as if a wasp had bitten me (…) it's crazy*!” (I.Q.)

***Acquisition of rubber hand features.*** Note that the previous descriptive statement could be classified as either acquisition of rubber hand features or attribution of real hand features to the rubber hand: in the first case, because the participant felt her hand to be swollen, assimilating the size of the rubber hand, and in the second, because this change in size was associated with swollenness due to a wasp bite, which is an event that can only occur in a living hand.

***Feeling of owning a prosthesis.*** One participant described a particular type of illusion that did not involve the experiential categories found for the illusion in the synchronous condition: instead he described an unusual illusory experience that somehow involved ownership of the rubber hand.

“*It's as if there were a prosthesis, really as if the prosthesis were my hand”* (G.R.)

#### Phase 2': absence of the illusion

In this phase the participants recognized the absence of feelings of identification or ownership toward the rubber hand.

***Absence of feeling of owning the rubber hand.***

“*You don't manage to let yourself be surprised, to say that it could be your hand* (…) *I really don't feel anything”* (V.C.)

***Feeling of ownership of the real hand.***

*Location of the tactile stimuli with respect to the hand*.

**“And toward the fake hand? Is there something or not?**

No, I, *well I have the sensations in the real hand”* (D.L.)

*Global location of the hand*.

“I don't at all have the impression that the fake hand is becoming mine actually. So actually I distinguish them clearly, *I know very well where my hand is actually*, no association” (H.M.)

## Discussion

The main objective of this study was to carry out a fuller examination of the phenomenology of the RHI, including the different stroking conditions involved in this experimental paradigm. More specifically the objectives were: (a) to assess whether the different stroking conditions present in the RHI protocol trigger ownership of the rubber hand and/or disownership of the real hand; (b) to investigate whether the RHI involves a transfer of both the passive and the active components of touch or if the two are dissociated; and (c) to identify the stages of the emergence and evolution of the illusion in the participants' experience.

### Feeling of ownership and disownership according to the different stroking conditions

#### Synchronous condition

The results showed that the illusion induced by synchronous stroking of the rubber and real hands involved a series of experiential categories: “feeling of ownership of the rubber hand,” “disregarding the real hand,” “delocalization of the real hand,” “disruption of proprioception,” “attribution of real hand features to the rubber hand,” and “acquisition of rubber hand features.”

One of the objectives of this study was to determine whether the illusion involves disownership of the real hand in addition to the feeling of ownership of the rubber hand. But, how do we identify disownership? In the present context, disownership refers to the absence of the real hand from participants' experience and/or the removal of the real hand from participants' internal body representation. This could be assessed, for instance, by participants' descriptions of “disregarding” their real hand, which in fact some participants did describe doing. However, if the participants did not state explicitly and spontaneously that they had forgotten their real hand, a problem arises when trying to determine whether the real hand was “absent” from their experience. One way to get around this problem is to consider a lack of descriptions regarding the real hand as an indicator. However, a lack of description does not necessarily indicate an absence of experience. In addition, considering that in the RHI attention is oriented to the rubber hand, participants' spontaneous reports generally concerned not their real hand but the rubber hand. One way to try and resolve this difficulty is by asking participants about their feelings toward their real hand: the problem in this case, however, is that the fact of inquiring about their real hand normally reminds participants of its presence as part of their bodies.

Therefore, in order to determine whether participants experienced disownership of their real hand, we could also consider indirect indicators of disownership, such as participants' descriptions of the misplacement of their real hand. We considered that the experiential categories “disregarding the real hand,” “delocalization of the real hand,” and “disruption of proprioception” suggested that the illusion involved disownership of the real hand as well as ownership of the rubber hand.

These results are in line with the view that the RHI involves a replacement of the real hand by the rubber hand (e.g., Armel and Ramachandran, 2003; Ehrsson et al., 2007; Moseley et al., 2008a; Tsakiris, 2010) and go against the view of Schütz-Bosbach et al. (2009) who argued that there need be no real disownership or replacement of the participant's hand and that the rubber hand could simply be incorporated into the body schema, in addition to the real hand.

Lewis and Lloyd (2010), who also investigated the subjective experience of the RHI, identified a component that they labeled as “change in the location of the hand.” Their category is equivalent to our “delocalization of the real hand,” since both refer to descriptions of a disruption in the ability to localize the real hand. In addition, our results suggest that the RHI can affect the ability to localize one's own hand with respect to different and possibly dissociable points of reference: the location of the hand in relation to tactile stimulation from the paintbrush; the location of the hand in relation to contact with the table; and the location of the hand in relation to the rest of the body.

#### Asynchronous condition

Among the numerous studies that have used the RHI as an experimental paradigm, the asynchronous stroking condition is the most frequent control condition, considered neutral in terms of transferring ownership to the rubber hand. But seven out of ten participants in our study experienced the illusion in the asynchronous condition; importantly, two of them did not experience the illusion in the synchronous condition. This result reveals a possibility that has previously been disregarded. Our study shows that the asynchronous condition was not neutral for seven out of ten participants, and that it also can give rise to a very peculiar experience. These results confirm those of Lewis and Lloyd (2010), who found that 84% of their participants experienced ownership in the asynchronous condition.

The experiential categories that characterize the illusion in the asynchronous condition involve (a) feeling of ownership of the rubber hand, (b) feeling of ownership of the real hand, (c) attribution of real hand features to the rubber hand, (d) feeling a phantom sensation, and (e) feeling a numbed hand.

A peculiarity of the illusion in the asynchronous condition is that participants felt ownership of the rubber hand and at the same time they felt the presence of their own hand. Contrary to the synchronous condition, in the asynchronous condition there was no disregarding of the real hand: even when participants could feel ownership of the rubber hand, they still felt that their hand was there. However, in some cases participants were confused regarding the localization of the tactile stimulation and the localization of their hand: they could not determine whether they felt the tactile stimulation on their hand or on the rubber hand. Interestingly, they could determine that they felt the contact with the table *underneath* their hand. This result suggests that while the passive aspect of touch is delocalized toward the rubber hand, the localization of the active aspect of touch in the illusion is not modified by the asynchronous stroking condition.

Another peculiarity of the illusion in the asynchronous condition is that some participants felt what we called “phantom sensation.” Although these participants realized that there was no correspondence between the visual and tactile stimulations, some of them had the impression of feeling on their hand what they saw happening on the rubber hand. In two cases there were moments at which the participants were not even able to say whether there was an asynchronicity; in other cases the participants felt tactile stimulation where they saw the paintbrush touching the rubber hand, but more faintly. Lewis and Lloyd (2010) also observed this phenomenon and this result is also in line with a finding reported by Durgin et al. (2007) in which 61% of participants stated feeling a sensation (thermal or touch) on their own hands when looking at a laser beam projected onto a rubber hand.

This effect might be related to the referred sensations described by Schaefer et al. (2006a,b) in a modified paradigm of the RHI. In Schaefer et al. (2006b), participants were stimulated on their little finger while watching a video showing a life-sized hand where the index finger was stimulated. There were two conditions: one in which the stroking of the real hand and the stroking shown in the video were in phase and another condition in which they were out of phase. Neuromagnetic source imaging of the topography of the functional organization of primary somatosensory cortex (SI) was recorded in order to investigate whether referred sensations were related to a change in activation pattern of SI. The results showed that participants reported feeling a tactile sensation on the index finger rather than on the little finger in the in phase condition. In addition, the extent of the cortical representation of the little finger increased during the illusion. The authors suggested that the conflict between vision and somatosensation results in a referral of the perceived sensation and that this illusion is related to short-term plasticity in SI.

In our experiment, the asynchronous condition corresponded to stroking the rubber and the real hand at different places at a given moment, which is similar to the in phase condition of Schaefer et al.'s experiment. The combination of the expectation of feeling a tactile sensation at a given place and the conflict between vision and somatosensation might have resulted in the described phantom sensation.

This experiential category is unique to the asynchronous condition, and it suggests that although the effects are different from those in the synchronous condition, the asynchronous condition can produce another strong illusion: the illusion of feeling something where physically there is no tactile stimulation.

In addition, two participants described having the impression of owning a “numbed” hand at the location of the rubber hand. One of them, who did not experience the illusion in the synchronous condition, explained that this feeling of having a numbed hand brought him closer to having the experience of owning the rubber hand. The impression of having a numbed hand involves a distinct mode of appropriation of the hand that was identified only in the asynchronous condition.

These results, and specifically the occurrence of these last two experiential categories, show that asynchronous stroking can induce a very particular and complex type of illusion.

#### Non-stroking condition

Three out of ten participants experienced an illusion in the non-stroking condition. In two of these cases, the experience of the illusion shared most of the characteristics already mentioned for the illusion in the synchronous condition. One participant's illusion was peculiar: he reported his experience of the incorporation of the rubber hand as being like having a prosthesis. As in the case of feeling a numbed hand in the asynchronous condition, we treated this experience as a distinct mode of appropriation of the hand, and although it has different characteristics than the illusion in the synchronous condition, it too involved a complex illusory experience.

In summary, these results show that both the asynchronous stroking condition and the non-stroking condition can induce an illusion that, in some cases, involves a highly complex experience. The number of participants who had some kind of illusory experience in the asynchronous and synchronous conditions was almost the same. Strikingly, two of the participants who experienced the illusion in the asynchronous condition did not experience it in the synchronous one. In the non-stroking condition considerably fewer participants experienced illusory sensations than in the other two conditions. In contrast to the illusion in the synchronous condition, the illusion in the asynchronous and non-stroking conditions did not involve the disregarding of the real hand: participants who experienced an illusion in these conditions felt that the rubber hand belonged to them but at the same time experienced the presence of their real hand. The presence of the real hand was expressed through their ability to locate it in space. However, in some cases (mainly in the asynchronous condition), the participants' experience of the illusion was characterized by a confusion about the location of the tactile sensation. Importantly, situations where the illusion was absent were also characterized by the ability to localize their hand in space. These results suggest that the experience of “owning” one's body parts is tightly linked to the ability to localize them. We observed that the localization of one's own body parts can involve different frames of reference: in relation to dynamic tactile stimulation on that part, in relation to its contact with an external surface, and in relation to the rest of the body. The contributions of these different points of reference to the experience of ownership remain to be assessed.

### Touching vs. being touched

The results showed that for the synchronous condition, in Phase 1 some participants described feeling the tactile sensation produced by the paintbrush on the rubber hand while all of them described feeling the tactile sensation produced by the contact with the table under their real hand suggesting that at this stage, only the passive aspect of touch is delocalized to the rubber hand. In Phase 2, all participants felt the tactile sensation produced by the paintbrush on the rubber hand and they also described feeling the contact with the table under the rubber hand, suggesting that at this stage both the passive and active aspects of touch are delocalized to the rubber hand. In Phase 2′, which corresponds to the absence of the illusion, neither the active aspect of touch nor the passive aspect were transferred to the rubber hand.

In the case of the asynchronous stroking condition, the sub-phase 5 of Phase 1 indicates that there was a confusion regarding the location of the tactile sensation produced by the paintbrush and of the location of the arm with respect to the rest of the body. Contrary to what happened in the synchronous condition, in the asynchronous one, participants felt the tactile sensation of the contact with the table under their real hand. This result suggests that the illusion in the asynchronous stroking condition differs from the illusion in the synchronous stroking condition, in the sense that the illusion does not involve a delocalization of the passive aspect of touch, but a “confusion” of its localization. In addition, the illusion in the asynchronous stroking condition does not involve delocalization of the active aspect of touch.

In the non-stroking condition, participants who experienced the illusion described projecting the tactile sensation produced by the paintbrush onto the rubber hand, which could be interpreted as a type of delocalization of the passive aspect of touch toward the rubber hand. Two participants also described feeling the contact with the table under the rubber hand during the illusion. Surprisingly, despite the fact that in the non-stroking condition participants do not see the rubber hand being stroked, concerning the passive and active aspects of touch, the illusion shared similar characteristics to that elicited by synchronous stroking.

It is important to highlight that in most cases, participants' spontaneous reports described the transfer of the passive component of touch (the location of the tactile stimulation) rather than the active component (the location of the contact with the table). Only one participant spontaneously reported feeling contact with the table under the rubber hand; in the remaining cases, this was assessed through questions. The lack of spontaneous reports regarding the active component of touch might be due the characteristics of the experimental protocol. We will come back to this issue in the “Limitations and future directions of research” section at the end of the article.

In summary, if we consider the illusion elicited by the synchronous stroking condition as the standard illusion, the result indicates that the passive aspect of touch was transferred at earlier stages of the illusion, and that the active aspect of touch was transferred only once the illusion was well established. This result might suggest a dissociation between these two aspects, and that the active aspect of touch is more “resistant” to the illusion. This opens up the hypothesis that the feeling of body ownership is more strongly related with the active dimension of touch than with the passive one.

### Emergence and evolution of the illusion

The analysis of the interviews allowed identifying the generic diachronic structure of each stroking condition. These structures were composed by two main phases and a series of sub phases that account for the microgenesis of the illusion, or for the absence of it.

The results showed that three sub-phases lead to the illusion in the synchronous stroking condition. The association between the visual and the tactile stimulation appears to be a key step for the emergence of the illusion since the two participants who, after establishing this association identified an incongruence between the tactile and visual stimuli, did not experience the illusion.

The existence of two phases, one for establishing the visuo-tactile association, and another for the illusion itself, is in line with what has been found at the neural level. Evidence has shown that activity in parietal and premotor cortex during the RHI might be related to the multi-sensory integration that causes the illusion, and that the activity of the posterior insula and operculum is related to the sense of owning the rubber hand (Tsakiris et al., 2007a).

These results partly confirm those of Lewis and Lloyd (2010), and also expand on them. Lewis and Lloyd described an evolution of the experience of the illusion in the synchronous condition. Here, not only did we find an evolution of the emergence of the illusion that may warrant further investigation—in addition we described the emergence of the experience of illusion in the asynchronous and non-stroking conditions.

Lewis and Lloyd (2010) argued that there is an ordered progression of perceptual events over the course of the illusion. Although we found similar perceptual events, we were not able to identify a generic ordered progression within the phases of the illusion. They wrote that the first event in the progression is the participants' report of feeling a rubbery texture on their hands. This feeling is similar to what we identified as the acquisition of rubber hand features. Second, they said, comes ownership of the rubber hand or the feeling that the real hand has moved to the location of the rubber hand. These components are equivalent to what we identified as the feeling of ownership of the rubber hand and the disruption of the ability to localize the real hand. Next, they wrote, come feelings of agency and wider bodily changes. These components are similar to what we identified as the attribution of features of the real hand to the rubber hand. However, in our analysis we were not able to establish a generic order of appearance of the different components that characterize the illusion. The method used by Lewis and Lloyd to establish the order of the components was to look at the time at which the description referring to the specific component appeared in the interview. But as shown by other studies using interviews (Jahn, 2005), the order of descriptions does not always correspond to the order in which the experience was lived. We reorganized participants' description according to the order in which the experience unfolded, and not according to the order in which the experience was described (see Petitmengin, 2006; Valenzuela-Moguillansky, 2012). Despite the fact that this allowed us to identify the sequence of sub-phases of Phase 1 for each stroking condition, we could not identify an ordered progression in the appearance of the experiential categories that characterized Phases 2 and 2'.

Interestingly, our results indicate that the illusion can be elicited in the asynchronous and non-stroking conditions, even though participants recognize the incongruence between the visual and tactile stimuli in these conditions. In these cases, the micro-genesis of the illusion seems to be more complex since more sub-phases were identified. In the asynchronous condition, the recognition of the visuo-tactile incongruence leads, in some participants, to a confusion of the location of the tactile sensation elicited by the paintbrush. Participants who concentrated on the visual input of the contact between the paintbrush and the rubber hand rather than on the tactile sensation elicited by the paintbrush on their real hand experienced the illusion, suggesting that concentrating on the visual input is a key step for the emergence of the illusion. In the non-stroking condition, after recognizing the incongruence between the visual and tactile stimuli, three participants projected the tactile sensation elicited by the paintbrush onto the rubber hand. Two of them described that concentrating on this sensation lead to the illusion, suggesting that this sub-phase also had a key role for inducing the illusion in the non-stroking condition.

In the pilot experiment that we conducted with individuals who were trained in the elicitation technique, one participant described in a clear way the impact of his mode of attention on the emergence of the illusion in the synchronous stroking condition. The participant's description suggests that, in the early stage of the stroking period, when he did not feel yet that the rubber hand belonged to him, the mode of attention was “global” and his field of vision was large, as illustrated by the following extracts of his interview:

“*I focused on the hand in a global manner*, (…) *everything was … uh … it was captured by my attention in a global way, not in a focused way.”*

“I have this hand here in my field of vision (his left hand, which is approximately 20 cm away to the left of the rubber hand),(…) I see that hand (the rubber hand), and the movement of the brush.”

“but I still feel the brush here, (indicating his right hand)… I feel it pretty well.”

Then, the participant's description suggests that there is a narrowing of his mode of attention and of his visual field that accompanies the emergence of the illusion:

*“And then after a while this field of vision gets narrow. It shrinks* (…) *my visual attention is focused on the brush… Everything was blurred around … and I felt the illusion happen.”*

These descriptions suggest that there is a change in the mode of attention and a narrowing in the visual field that facilitates the emergence of the illusion and perhaps its maintenance. To our knowledge previous studies have not assessed the role of attention in the emergence and modulation of the illusion.

### Limitations and future directions of research

A clear limitation of our study is that the small number of participants prevents the generalization of these findings to a large population. In addition, due to the small number of participants, we could not assess the influence of the order of the conditions in the presence of some experiential categories such as the “phantom sensation.” As stated in the results section, this experiential category was present in participants who underwent the asynchronous stroking condition after the synchronous stroking condition. In the synchronous stroking these participants experienced the feeling of ownership of the rubber hand as well as disownership of the real hand, determined by the experiential categories “disregarding of the real hand,” “delocalization of the real hand,” and “disruption of proprioception.” Using the term employed by Lewis and Lloyd (2010) this corresponds to a *recalibration of the body schema*, which might involve a recalibration of the location where the tactile sensation is expected to be. This expectation might have contributed to the presence of the phantom sensation.

However, it is important to stress that the validity of the results obtained through the elicitation interviews depends on the quality of the descriptions rather than on their quantity. This difference between first and third-person approaches gives rise to different validation frameworks, making it often difficult to integrate these two approaches. We believe that the present study is a step toward this integration and that further work needs to be done in order to assess all the methodological as well as epistemological issues that this integration raises.

Regarding the dissociation between the active and passive aspects of touch, as stated in the discussion, participants did not spontaneously report the transfer of the active component of touch. A limitation of our study's account of this dissociation is that the active part of the experimental manipulation in the RHI protocol takes place in the interaction between the paintbrush and the hand; no manipulation occurs at the interface between the table and the hand. Therefore, it is likely that the movements of the paintbrush directed the participants' attention to what was happening on the dorsum of their hand, while what was happening on their palm and under their fingers was outside of their focus of attention. A protocol that controls for this asymmetry by eliciting a redirection of the participant's attention toward the interaction between the table and the hand, combined with interviews oriented to investigate this facet of experience, are needed to further investigate the question of whether the active and passive aspects are differently affected by the illusion.

A future direction of research concerns the role of the mode of attention in the emergence and evolution of the illusion. Future interviews could aim at more systematically eliciting descriptions of the participants' mode of attention in the different phases of the illusion, in order to try to identify the impact of attentional modulation on the emergence of the illusion. Furthermore, a RHI protocol that combines this interview technique with methods that assess the neural correlates of attention could help to clarify the interactions between attention and the feeling of owning the rubber and the real hand as well as contributing to establishing the duration of the different phases of the illusion. Further study of the impact of the mode of attention on the emergence and permanence of the illusion could shed light on the role played by the mode of attention in the sense of body ownership. Studying the relationship between attention and the sense of body ownership appears very relevant for a better understanding of pain-related disruptions in body awareness. In a recent study Valenzuela-Moguillansky (2013) described the changes experienced by fibromyalgia patients over the course of a pain crisis. Patients' descriptions indicate that focusing attention on their bodily sensations is followed by a disruption of the perceived size of their painful body parts and a loss of the sense of body ownership.

Our results also have implications for the design of future experiments involving the RHI. First of all our results suggest that for an experimental protocol that attempts to assess the effect of the sense of body ownership on any dependent variable, the non-stroking condition appears to be a better control condition than the asynchronous stroking condition. Second, inter-individual variability of the subjective experience of the illusion should be considered when designing the protocol and analyzing and interpreting the results. This could be done by integrating a first-person approach in a first stage of the protocol to establish a typology of participants, for instance, in relation to the presence or absence of the illusion in each stroking condition (see Valenzuela-Moguillansky, 2012). Taking as example the results of the present study, the participants can be clustered into four groups: “as expected”—the participants who experienced the illusion in the synchronous stroking condition and who did not experience it at all in the asynchronous and non-stroking conditions; “not as expected”—the participants who did not experience the illusion in the synchronous condition but who experienced some illusory aspects in the asynchronous condition; “susceptible to the illusion,”—the participants who experienced the illusion in both the synchronous and asynchronous conditions; and “very susceptible to the illusion”—the participants who experienced the illusion in all three stroking conditions. In this way, the hypotheses would not be conceived in terms of the stroking conditions but in terms of the typology of participants. Accordingly, the impact of the illusion should not be assessed by averaging the data of a given dependent variable by condition, but considering this typology.

This approach of course presents some challenges. First, as the division of the participants by *types* would reduce the number of participants per group, the study should involve a large number of participants in order to have statistically significant results. Second, the integration of a first-person approach involves the inclusion of a first phase of interviews and their analysis in order to establish the typologies. However, the experience that participants have of each stroking condition might be different from the one in the actual experiment. Still, the prospect of designing experiments that consider the complexity of the experience that they claim to assess is worth the effort of facing these challenges.

## Conclusion

In summary, the use of a first-person approach, particularly the elicitation interview, allowed us to investigate and reveal aspects of the RHI such as the richness of the experience elicited by the different stroking conditions, and the presence of different phases in the genesis of the illusion which are usually concealed by the focalization on the final content of the illusion (Petitmengin and Lachaux, 2013). These had not previously been highlighted through the use of questionnaires. These findings enable us to refine both our understanding of the components of the feeling of body ownership and also the microdynamics of constitution of this feeling.

The basic implications of the results of the present study are, first, that the illusion induced by the synchronous stroking condition involves ownership of the rubber hand and disownership of the real hand, meaning that the illusion probably involves a replacement of the real hand by the rubber hand in participants' body schema, and not an addition of a third limb. Second, the study shows that the feeling of owning the rubber hand can be induced not only by synchronous stroking but also by asynchronous stroking and, to a lesser extent, in the non-stroking condition. In addition, the illusions induced by the asynchronous and non-stroking conditions have their own characteristics, which probably explains why they are not highlighted by the classic questionnaire used in experiments involving the RHI. Third, the cases of presence and absence of the illusion showed that the experience of hand ownership seems to be tightly linked to the ability to localize the hand in space. Fourth, we could identify different phases and sub-phases in the micro-genesis of the illusion that indicate that the passive aspect of touch was transferred at earlier stages of the illusion, and that the active aspect of touch was transferred only at a later stage. Finally, our findings are relevant at a methodological level. Taking into account the fact that the different stroking conditions can elicit different types of illusions, and that their emergence involves different phases, modifies the way in which an experimental protocol involving the RHI should be designed, and the way its results should be analyzed and interpreted.

A better understanding of the sense of body ownership might have clinical implications. In recent years, several studies have shown a relationship between chronic pain conditions and a disruption in different aspect of body awareness (e.g., Schwoebel et al., 2001; Förderreuther et al., 2004; Altschuler and Hu, 2008; Moseley et al., 2008b), particularly in the sense of body ownership (e.g., Galer and Jensen, 1999) and in the ability to localize the painful body parts (e.g., Sumitani et al. (2007). However, so far the mechanisms underlying this relationship are not well understood. Gaining understanding in the emergence of the sense of body ownership and its relationship with the ability to localize one's own body in space might contribute to better understand the relationship between chronic pain and disruptions in body awareness. Finally, in more general terms, a better comprehension of the components of body ownership and its micro-dynamics are crucial to advance the understanding of the sense of self, issue which is today at the core of many of the questions of cognitive science.

### Conflict of interest statement

The authors declare that the research was conducted in the absence of any commercial or financial relationships that could be construed as a potential conflict of interest.

## Footnotes

^1^Interviews were conducted in French; all the extracts of interviews reported in this article were translated into English by the authors.

^2^In French, the name of this technique is “Entretien d'explicitation.” It was first translated to English as “Explicitation interview.” This year it was agreed that the English translation would be “Elicitation interviews.”

## Appendix

### Analysis of the elicitation interviews

The analysis of the interviews involves a recursive process of successive operations of abstraction and comparison. In what follows we will explain how we identified a generic structure of the experience in each visuo-tactile condition using the gathered data. The method that we used was based on the analytical methodology proposed by Petitmengin (1999, 2001).

#### Transcription of the interviews and division of the text

The first step of the analysis involved transcribing the interviews and numbering the lines of the transcription. This makes it possible to return to the original data once the extracts of descriptions are chosen and reorganized. After reading the interview, we divided the text in the descriptions according to each of the stroking conditions.

#### Identification and selection of the relevant part of the text

Different types of information compose interviewees' discourse, and only part of this information is considered in the analysis. The analysis component of the elicitation interview method suggests selecting only the fragments describing the interviewee's sequence of actions and the qualitative aspects of their experience, leaving aside information such as the context in which the experience occurs, or representations that we have about it, or theoretical knowledge related to the experience, which are considered “satellite information” (Vermersch, 2011). The selected fragments, called “descriptive statements,” are factual descriptions, as non-interpretative as possible, that refer to a particular experience and that are produced while in a state of evocation (Vermersch, 2009). Evocation refers to recalling a given experience as if re-enacting it. Because in the present experiment many of the descriptions were “in real time,” in addition to the descriptive statements, “in real time” descriptions that did not contain satellite information were also selected.

#### Identification of underlying structure in the experience

Once the relevant content of the interview was extracted, we proceeded to identify the underlying structure of the experience. By structure we refer to “a network of relationships between descriptive categories, independent of the experiential content,” as defined by Delattre (1971).

***First stage in the establishment of the experiential categories in individual experiences.*** Once we had selected the relevant part of the text for each interview, we identified descriptive statements referring to qualitative features of the experience and we grouped them according to different “experiential categories.” An experiential category is a class that groups together descriptive interview extracts that have a relevant aspect in common. This process of abstraction is called “instantiation.” For instance, from the following extracts, two descriptive statements which were classified under the experiential category “feeling of ownership” are marked in italics.

- “I don't have the impression of a transfer of the hand, but *directly that it's this hand that's mine, the fake hand”* (V.C.) - “Because *I really really have the impression that it [the rubber hand] belongs to me”* (V.C.)

This stage represents a first step in organizing the data; the established experiential categories changed during the following steps of analysis. After grouping and labeling all the descriptive statements from each interview, we proceeded to reorganize the description.

***Reorganization of the text.*** In the cases of verbalizations that were not produced “in real time,” the order of the description often did not correspond to the order of the lived experience, thus it was important to reorganize the text according to the chronology of the experience. In some cases, this is due to the fact that initially the interviewee gave a global outline of the situation and then, during the interview, different parts of the experience were explored in more detail. In other cases, the interviewee might have initially described aspects of the experience that were more salient to her and then, in later stages of the interview, pre-reflective aspects might emerge. For instance, one participant first referred to the impression that the rubber hand belonged to her. This impression was what first popped out in her experience. But then, as the interview continued, she referred to a more subtle aspect of her experience that took place at the beginning of the stroking period: her internal attitude of putting herself in a state of having no expectations regarding the experiment.

***Identification of the diachronic structure of an individual experience.*** Once the identified experiential categories were organized in sequential order, we proceeded to establish the diachronic structure of individual experiences. The diachronic structure is the set of different phases in the evolution of the experience. In order to do this, we looked for the participant's “inner gestures” or for markers that indicate a change in the participant's perception. Continuing on with the previous example, from the descriptive statements selected for participant V.C., we identified inner gestures and changes in perception that led to the establishment of the following two phases of the experience of the synchronous stroking. We gave each phase a name or label, usually on the basis of the descriptive statements:

Phase 1: “Association of visual and tactile stimuli”

Descriptive statements:

“I completely associate what I see with what I feel.” (124)^3^ “My sensation is exactly the same as what I see” (122)

In this case the inner gesture would be “I associate.”

Phase 2: “Illusion”

Descriptive statements:

- “Because [I really really have the impression that it] the rubber hand belongs to me too” (147) - “It's pretty bizarre because I forget that I have this right hand.” (142) - “They become unified, almost as if it were the same hand, but united in two, it's really, it's fairly bizarre” (141)

In this case, rather than an inner gesture, what defined the phase was a change in the participant's perception regarding the rubber hand. The change in perception was the impression of owning the rubber hand.

***Second stage in the establishment of the experiential categories: identification of the synchronic structure of an individual experience.*** The identification of the synchronic structure refers to the recognition of the different components that characterize the interviewee's experience in a given phase. It is like “zooming in” on a particular phase, to identify and categorize the qualitative aspects that give its identity.

At this stage, through processes of “aggregation” and “generalization” we grouped together the previously identified experiential categories into experiential categories of a higher order of abstraction. Aggregation refers to “an operation of abstraction through which a relationship between objects is regarded as a higher-level object” (Smith and Smith, 1977). On the other hand, generalization refers “to an operation of abstraction in which a set of similar objects is regarded as a generic object” (Smith and Smith, 1977). For instance, in the example given above, the “illusion” phase contained three descriptive statements: (147), (142), and (141). In the establishment of the synchronic structure of this phase, each of these statements was classified through a process of aggregation under the three experiential categories: “feeling of ownership of the rubber hand,” “disregarding the real hand,” and “impression of unification of the real and rubber hand.”

The aggregation and generalization processes can be ascendant or descendent (Petitmengin, 2001). The categorization is *ascendant* when we start from the text and progressively establish categories at a higher and higher level of abstraction. The categorization is *descendant* when we predefine the categories and classify the text according to the chosen definition. In the case of the present study, some of the experiential categories were classified through an ascendant categorization and others through a descendant categorization. For instance, as explained above, some questions were pre-established based on pilot experiments. These questions led to the emergence of experiential categories through a descendent process. Others, such as “disregarding the real hand,” were identified through an ascendant process. In some cases, categories that emerged from the participants' descriptions were used as guides for later interviews. For instance, as explained in the introduction to the article, the distinction between the felt position of the contact of the paintbrush on the hand (rubber or real) and the felt position of the contact of the hand with the table (under the rubber or the real hand) emerged from the experience of one participant and thereafter was systematically assessed in the interviews. In these cases, subsequent descriptions regarding this distinction were categorized according to a descendent classification.

***Describing the general structure of the experience of each visuo tactile condition.***

***Comparison of the individuals' diachronic structures.*** The aim of the description of the generic diachronic structure is to identify the invariant among the different experiences by comparing the diachronic structures of individuals.

In order to do so, we grouped together the descriptive statements we would use to identify the landmarks of each phase in the individual experiences and also the labels of each phase of the individual experiences. We then determined the invariants and possible variants of the experiences

We identified two possible criteria for selecting the final generic structure: an “exclusive” and an “inclusive” criterion. The exclusive criterion would select only the phases that appeared most frequently in participants' descriptions. The inclusive criterion would consider all the phases identified in the participants' descriptions. As the character of the present study was exploratory, and the participants had different levels of expertise in describing their experience, we decided to adopt an inclusive criterion in order to consider infrequent aspects of the experience for future inquiry.

The following is an example of the generic diachronic structure identified in the individual structures of participants C.G, H.M., C.V., M.W, and, I.P.:

**C.G.**

Phase 1: Recognition of correspondence between the visual and tactile stimuli

Sub phase 1: Feeling the tactile sensation of the paintbrush

Sub phase 2: Association of the visual and tactile stimuli

Sub phase 3: Beginning of rubber hand incorporation

Phase 2: Illusion

**H.M.**

Phase 1: Recognition of correlation between the visual and tactile stimuli

Sub phase 1: Association of the visual and tactile stimuli

Sub phase 2: Beginning of rubber hand incorporation

Phase 2: Illusion

**C.V.**

Phase 1: Recognition of correlation between the visual and tactile stimuli

Sub phase 1: Association of the visual and tactile stimuli

Sub phase 2: Beginning of rubber hand incorporation

Sub phase 3: Violation of expectation

Phase 2': Absence of the illusion

**M.W.**

Phase 1: Recognition of correlation between the visual and tactile stimuli

Sub phase 1: Beginning of rubber hand incorporation

Sub phase 2: Violation of expectation

Phase 2': Absence of the illusion

**I.P.**

Phase 1: Recognition of correspondence between the visual and tactile stimuli

Sub phase 1: Feeling the tactile sensation of the paintbrush

Sub phase 2: Association of the visual and tactile stimuli

Sub phase 3: Beginning of rubber hand incorporation

Phase 2: Illusion

Comparing the diachronic structure of these participants in the synchronous stroking condition, we observe that G.G. and I.P.'s sub phase 1 is not present for the other three participants, that sub phase 2 for G.G. and I.P and Phase 1 for H.M. and C.V. seem to be equivalents, and that the presence of sub phase “violation of expectation” in participants C.V. and M.W. leads to Phase 2'.

Thus, a generic (inclusive) diachronic structure here would be a) In the case that the illusion is elicited:

Phase 1: Recognition of correspondence between the visual and tactile stimuli

Sub phase 1: Feeling the tactile sensation of the paintbrush

Sub phase 2: Association of the visual and tactile stimuli

Sub phase 3: Beginning of rubber hand incorporation

Phase 2: Illusion

(b) In the case that the illusion is not elicited:

Phase 1: Recognition of correlation between the visual and tactile stimuli

Sub phase 1: Feeling the tactile sensation of the paintbrush

Sub phase 2: Association of the visual and tactile stimuli

Sub phase 3: Beginning of rubber hand incorporation

Sub phase 4: Violation of expectation

Phase 2': Absence of the illusion

***Comparison of the individuals' synchronic structures.*** Once the individuals' descriptions were divided into phases and a generic diachronic structure was identified, we compared the experiential categories that characterized each phase for each participant. Again through a process of aggregation and generalization, experiential categories of similar class were grouped together under experiential categories at a higher level of abstraction.

Like the establishment of the generic diachronic structure, the establishment of the generic synchronic structure was inclusive, meaning that all the experiential categories that had been identified in the specific experiences were taken into account in the generic structure, even if they had appeared only in one experience.

For instance, the illusion phase of C.G., I.Q., H.M. and D.L. was characterized by the following experiential categories, respectively:

**C.G**.

Feeling of ownership of the rubber hand

Acquisition of rubber hand features

Losing ownership of the real hand

**I.Q.**

Feeling of ownership of the rubber hand

Attribution of real hand features to the rubber hand

Disregarding the real hand

**H.M.**

Feeling of ownership of the rubber hand

Feeling only the rubber hand

**D.L.**

Feeling of ownership of the rubber hand

Acquisition of rubber hand features

Feeling only the rubber hand

Delocalization of the real hand

**I.P.**

Disruption of the ability to determine the position of the real hand

Feeling only the rubber hand

Acquisition of rubber hand features

Attribution of real hand features to the rubber hand

Thus, after the processes of comparison, aggregation and generalization, the generic synchronic structure of the illusion phase was:

– Feeling of ownership of the rubber hand
– Disregarding the real hand
– Delocalization of the real hand
– Disruption of proprioception
– Attribution of real hand features to the rubber hand
– Acquisition of rubber hand features
